# Clinical-Functional Vulnerability of Older Adults in Primary Care in a Brazilian Municipality: Associated Factors

**DOI:** 10.3390/ijerph22101583

**Published:** 2025-10-18

**Authors:** Cleomar Ana de Souza Valentim, André Silva Valentim, Maria da Luz Rosário de Sousa, Marília Jesus Batista

**Affiliations:** 1 Department Community Health, Faculty of Medicine of Jundiaí (FMJ), Jundiaí 13202-550, SP, Brazil; 2 Department of Internal Medicine, Faculty of Medicine of Jundiaí (FMJ), Jundiaí 13202-550, SP, Brazil; 3 Department of Collective Health, Pediatric Dentistry and Orthodontics, School of Dentistry of Piracicaba, University of Campinas (FOP–Unicamp), Piracicaba 13414-903, SP, Brazil

**Keywords:** nutritional status, oral health, sarcopenia, frailty, elderly

## Abstract

Objective: The objective of this study was to assess clinical-functional vulnerability (CFV) and associated factors in community-dwelling older adults treated in primary care. Methods: A cross-sectional study was conducted with non-institutionalized elderly individuals ≥60 years randomly selected from five Health Units in Jundiaí/SP, Brazil, in 2023. Sociodemographic data, health behaviors, and data on oral health (number of teeth; chewing: good/fair/poor), cognitive function (10-CS), nutritional status (MNA), health literacy (HLS-14), sarcopenia (SARC-F+CC) and frailty (IVCF-20) were collected. Descriptive and bivariate analyses between the outcome (CFV) and the independent variables were performed using the chi-squared test and binary logistic regression models (*p* < 0.05). Results: A total of 211 older adults participated in this study; 72% were female and the mean age was 70.41 years (±7.45). Regarding CFV, a high risk was identified in 9.5% of the participants (*n* = 19), a moderate risk in 34.6% (*n* = 73), and a low risk in 55.9% (*n* = 118). After adjusting the regression model, the following variables were associated with CFV: lower income (OR = 1.90; 95%CI: 1.02–3.55), poor (OR = 5.18; 95%CI: 2.13–12.63) and fair (OR = 2.36; 95%CI: 1.10–5.05) chewing, risk of malnutrition or malnourished (OR = 2.36; 95%CI: 1.23–5.52), and low literacy (OR = 1.86; 95%CI: 1.09–3.45). Conclusion: Socioeconomic factors, nutritional status (underweight or malnourished), poor or fair chewing, and low health literacy were associated with CFV among older people. Strengthening primary health care through targeted interventions may help prevent frailty or delay its progression. Understanding the predictors of frailty can guide health professionals, managers, and researchers in designing preventive and health promotion strategies, as well as public policies within Primary Health Care.

## 1. Introduction

According to the World Health Organization (WHO), by 2030, one in six people will be 60 years or older [1]. Population aging is a global phenomenon. One of the problems observed with advancing age is the increase in the prevalence of frailty. The pre-frail condition is an intermediate stage between healthy aging and frailty syndrome. According to the ELSI-Brasil [2] study, in Brazil, 13.4% of older adults are frail and 54.5% are pre-frail, conditions that have a significant impact on the lives of this population, their families, health services, public health, and social assistance.

Frailty syndrome is an identifiable clinical condition characterized by a progressive decline in strength and resistance, as well as a reduction in different physiological systems, increasing the risk of health complications and leading to greater dependence, impairment in activities of daily living, loss of autonomy, hospitalization, and increased mortality [3]. It is worth noting that, although senescence and frailty are associated with chronological age, aging alone is not a determinant or predictive factor for the development of these conditions.

Some of the factors associated with frailty are reversible or preventable, such as social determinants of health and specific clinical conditions [4]. Important social determinants that influence aging include the social support network, socioeconomic factors, the environment where the individual lives, and lifestyle [5]. Health literacy (LH) is an ability to access, understand, and interpret health-related information in order to guide healthy choices [6]. Limited health literacy can contribute to a vulnerable health status, increasing the use of health services and resulting in unfavorable outcomes in different areas [7] such as oral health care, maintenance of an adequate diet, or daily life care.

Among the modifiable factors that influence frailty syndrome, nutritional status [8] and oral health [9] are intrinsically linked and draw attention because of their important role in the homeostasis of clinical conditions of aging. Nutrition is necessary to maintain the health of oral tissues [9], contributing to the maintenance of a sufficient number of teeth for adequate chewing [10]. Impairment of oral functionality can affect the adequate intake of macro- and micronutrients and consequent nutrient absorption, compromising nutritional status [8]. This nutritional imbalance can lead to the progressive loss of muscle mass and functional strength and consequent sarcopenia [11]. Comprehensiveness in healthcare requires actions that address adverse health-related conditions throughout life in order to improve the individual’s quality of life [12].

The Clinical-Functional Vulnerability Index (IVCF-20) was developed in Brazil and is a multidimensional screening instrument for the rapid identification of frail older adults with satisfactory reliability and sensitivity [13]. However, in addition to recognizing older adults as frail, pre-frail, or robust, it is crucial to identify the factors associated with clinical-functional vulnerability (CVF). Understanding the environment, habits, and social determinants of health makes a difference in health care and can reduce the impact on public health systems. The IVCF-20 is an easy-to-apply tool that can serve as a basis for the screening and identification of older adults with different levels of frailty in primary care [14].

Considering the continued rise in life expectancy, the early identification of older adults vulnerable to frailty and of the factors associated with this condition is essential for preventing adverse health outcomes. Although frailty has been widely investigated, few studies have explored its multifactorial determinants in community-dwelling older adults within the context of Primary Health Care (PHC) in Brazil using a multidimension instrument. Unlike most previous studies conducted in long-term care institutions (LTCIs), this research focused on non-institutionalized, community-dwelling older adults, providing a broader and more realistic view of aging and vulnerability in PHC setting. Therefore, the aim of this study was to assess clinical-functional vulnerability (CFV) and its associated factors among community-dwelling older adults assisted by the PHC services of the Brazilian Unified Health System (SUS).

## 2. Materials and Methods

This is a quantitative, analytical, cross-sectional study conducted from June to August 2023 in the municipality of Jundiaí, state of São Paulo, Brazil. According to data of the Brazilian Institute of Geography and Statistics (IBGE), the municipality has an aging index of 100.96% (1980–2021), with 18.63% of its population being composed of people aged 60 years or over [15]. The municipality has 35 Health Units; 10 of them employ the Family Health Strategy (FHS) and total Primary Health Care (PHC) coverage is 60.22% [16].

Sample size calculation was based on the prevalence of frailty of 13.4% obtained in the ELSI-Brasil study [2]. A significance level of 5% (α = 0.05), a design effect—deff of 2, and an error of 10% were adopted, resulting in a minimum number of 108 older adults. Considering a possible loss of 20%, a final sample size of 135 older adults was established. However, to ensure robustness of this study and to account for possible losses, an additional 40% was added to the sample, totaling 225 participants.

The sample was selected in two stages. First, five Basic Health Units (BHUs) located in different regions and with different sociodemographic profiles were selected by convenience, three employing Family Health Strategy (FHS) and two traditional BHUs. All units use prevention and health promotion strategies (Integrative health practices, sports activities, and public lectures). Next, a minimum of 45 individuals from each BHUs (n = 225) were randomly invited to comprise the sample. At the BHUs, data were collected from older adults who attended the unit for treatment/activities or by scheduling home visits conducted by community health agents.

The following inclusion criteria were adopted: community-dwelling older adults aged 60 years or over, who did not have cognitive impairment assessed by the 10-Point Cognitive Screener (10-CS) [17] and who were capable of expressing themselves verbally and undergoing the necessary tests. Individuals receiving palliative care, individuals with a diagnosis of neurodegenerative diseases such as Parkinson’s, Alzheimer’s, or other type of dementia of any etiology, and individuals with terminal illnesses such as cancer were excluded. Older adults with severe physical, visual, or hearing impairments that could compromise communication or prevent them from completing the questionnaire were also excluded. However, participants with mild visual or hearing impairments that did not interfere with their ability to understand or respond to the instrument were considered eligible.

The participants were invited to complete a questionnaire consisting of 106 objective questions. The questionnaire was applied by interview and lasted a maximum period of 50 min. The questionnaire was administered slowly and carefully, allowing each participant as much time as needed to respond, ensuring comfort and comprehension throughout the process. Cognitive function (10-CS), nutritional status (MNA), health literacy (HLS-14), sarcopenia (SARC-F+CC), and frailty (IVCF-20) were also assessed. Data were collected by one of the researchers, who underwent an 8 h training session with a standard examiner. The questionnaire consisted of the following items:

- Demographic data: age, sex (female, male), skin color (white, black/brown), marital status (single, married), education (incomplete or complete elementary school, high school, or higher), and income (up to 1 minimum wage, 2 or more minimum wages).
- Clinical conditions: weight (kg) measured with an Onron HBF-214 digital scale and height (cm) measured with a tape measure for the calculation of body mass index (BMI) the classification proposed by the Pan American Health Organization (PAHO/OPAS) recommended for older adults and considers age-related body composition changes: underweight (BMI ≤ 23 kg/m^2^), normal weight (23 < BMI < 28 kg/m^2^), overweight (28 ≤ BMI < 30 kg/m^2^), and obesity (BMI ≥ 30 kg/m^2^) [18,19]. Health information was self-reported: health problems (yes, no), pre-existing diseases (diabetes, hypertension, both, others), history of COVID-19 (yes, no), oral health variable measured by number of teeth, (number of teeth ≤19 or ≥20), and chewing ability (good, fair, poor). According to the World Health Organization (WHO) [20], the number of natural teeth is an established epidemiological indicator of oral health, with ≤19 indicating non-functional dentition and ≥20 representing functional dentition. Chewing ability was included as it reflects the functional variables of oral health, closely related to nutrition and overall well-being.
- Health behavior: previous medication use (no medication, 1 to 3 medications, 4 or more medications), as well as habits such as smoking, alcohol consumption, and physical activity (yes, no).
- Use of health services: hospitalization in the last year (yes, no), frequency of use of BHU (1x year, 2x or more per year) and participation in health promotion activities (yes, no).

The 10-CS [17] was used to measure cognitive function. This instrument consists of six questions covering temporal orientation, categorical fluency, and memory. The score ranges from 0 to 10 and is adjusted for educational level. A score ≥8 indicates normal cognitive function, a score between 6 and 7 indicates possible impairment, and a score of 0 to 5 indicates probable impairment.

Nutritional status was assessed using the Mini Nutritional Assessment (MNA) [21]. This instrument is composed of anthropometric measurements, dietary information, global assessment (lifestyle, medication, functional status), and self-assessment (self-perception of health and nutrition). A final score ≥24 indicates normal nutritional status, a score ≥17 to <24 indicates a risk of malnutrition, and a score <17 indicates malnutrition.

A Brazilian version of the 14-item Health Literacy Scale (HLS-14) [22] was used to assess health literacy. The scale consists of 14 questions answered on a Likert scale, with scores ranging from 14 to 70, and assesses literacy at the functional, critical, and communicative levels. The cutoff point for high and low literacy was defined at the median (with both high and low health literacy being considered).

Sarcopenia risk was assessed using the SARC-F+CC [23], which considers aspects such as muscle function and strength, in addition to calf circumference. There are different cutoff points for women and men. The score ranges from 0 to 20, with a score ≥11 suggesting sarcopenia.

Frailty was measured using the IVCF-20 [13], a validated and rapid screening tool designed to identify vulnerability or frailty in older adults within primary care settings. The IVCF-20 comprises 20 items that evaluate key predictors of functional decline, including age, self-perceived health, performance in activities of daily living, cognitive status, mood, mobility, communication abilities, and the presence of multiple comorbidities. Each item contributes one point to the total score, resulting in a possible range from 0 to 40. Based on the final score, individuals are categorized as follows: 0–6 points (low), older adults with a low risk of CFV; 7–14 points (medium), older adults with a moderate risk of CFV; ≥15 points (high), and older adults with a high risk of CFV. For the purposes of this study, the IVCF-20 score was dichotomized into low CFV (≤6 points) and medium/high CFV (≥7 points).

In the present study, a level of significance of 5% and power >80% were adopted. Descriptive statistical analyses were performed using frailty measured with the IVCF-20 as the outcome. Explanatory variables included sarcopenia, nutritional status, and the number of teeth of the participants, in addition to the independent variables.

In bivariate analysis, the chi-squared test was applied to evaluate the association between the outcome (low CFV or medium/high CFV), using low CFV as a reference in the logistic regression, and the independent variables. Variables with a *p*-value < 0.20 in this analysis were included in the binary logistic regression model and adjusted until the final model was obtained. In addition, odds ratios (OR) were calculated as a measure of association.

All participants agreed to their participation by signing the informed consent form. The Research Ethics Committee of the Faculty of Medicine of Jundiaí approved this study (CAAE: 63588022.0.0000.5412, Approval number 6.547.860). This study was conducted in accordance with the ethical and legal principles established by Resolution 466/12, which provides guidelines for research involving humans, as well as by Resolution 510/2016.

All anonymized data supporting the findings of this study are available from the Open Science Framework (OSF) at https://osf.io/rg75y/?view_only=3f6ea18761ff4e8ebc309568c3334e23 (accessed on 4 February 2025).

## 3. Results

A total of 212 older adults participated in this study (minimum of 45 per unit). However, one individual did not meet the inclusion criteria and was excluded, resulting in a final sample of 211 participants.

There was a predominance of women (72%, n = 152), a mean age of 70.41 years (±7.45), white skin color (75.4%, n = 159), incomplete elementary education (58.3%, n = 123), and income of up to one minimum wage (39.3%, n = 83). Only 10% of the participants (n = 23) did not use medications daily, while 89.6% (n = 189) had some health problems, 45.5% (n = 96) former smokers, and 36.5% (n = 77) reported a history of COVID-19 infection. Regarding oral health variables measured by number of teeth, 78.7% of the participants (n = 166) had ≤19 teeth and 37% (n = 88) had fair/poor chewing (Table 1). The data related to the IVCF-20 presented below Table 1, are interpreted according to the original scale in three categories, in which higher scores indicate greater vulnerability or frailty. Most participants were classified as robust.

The application of the IVCF-20 among older people receiving primary health care revealed a predominance of individuals aged 60 to 74 years, who generally reported good self-perceived health, independence in activities of daily living, and preserved cognitive function. Table 2 shows de IVCF-20 distribution which demonstrated a substantial proportion of participants presented depressive symptoms and mobility limitations, as well as multiple chronic conditions and the use of several medications. (Table 2).

Bivariate analysis of CFV identified the following variables as significant: age, skin color, marital status, education, income, medication use (divided into two categories: up to 3 medications/4 or more medications), health problems, use of BHU, number of teeth (≤19), chewing (fair/poor), SARC-F+CC (risk of sarcopenia), cognitive function (possible impairment), MNA (risk of malnutrition or malnourished), and health literacy (low) (Table 3).

Table 4 shows the results of univariate analysis and statistically significant associations after logistic regression. The following factors were associated with medium or high CFV: income up to one minimum wage (OR = 1.90; 95%CI: 1.02–3.55); poor (OR = 5.18; 95%CI: 2.13–12.63) and fair chewing (OR = 2.36; 95%CI: 1.10–5.05); MNA—risk of malnutrition or malnourished (OR = 2.36; 95%CI: 1.23–5.52), and HLS-14—low health literacy (OR = 1.86; 95%CI: 1.09–3.45).

## 4. Discussion

This study analyzed multiple factors that could influence CFV in older adults. The results showed that an income up to one minimum wage, low health literacy, poor chewing ability, and inadequate nutritional status were associated with a higher risk of CFV. These findings highlight the importance of a comprehensive health approach for older adults, with access to a multidisciplinary team (eMulti) in PHC considering both intrinsic and extrinsic stressors.

A systematic review and meta-analysis using data from 62 countries across the world revealed a prevalence of frailty in the community-dwelling population of about 12% [24]. Using the IVCF-20, 9.5% of older adults were found to be at high risk for CFV. Considering the medium- to high-risk stages of CFV, the importance of early identification of frailty is evident, which is a public health priority aimed at reducing the costs associated with this condition and promoting healthy aging in this population [25]. The IVCF-20 is indicated for global assessment of older adults as it covers various dimensions. The advantages of this instrument include its simple applicability and multidimensionality, which permit its use by professionals of a multidisciplinary team and support networks, as well as by geriatrics or gerontology specialists [12,13,14]. In the same sample previously analyzed by Valentim et al. [26], the use of FRAIL-BR instrument identified 24.6% of older adults as frail, 37.9% as pre-frail, and 37.4% as robust. These findings highlight the value of applying a standardized frailty screening tool, allowing for meaningful comparison across studies and populations.

Given the heterogeneity of the aging process, there is a need for multidimensional measures, including attention to oral health, which is closely linked to socioeconomic factors, particularly the limited access to care among this population [9]. According to the World Health Organization (2022), oral health is integral to overall health and includes the ability to chew and eat properly, both of which are essential for adequate nutrition and well-being. Functional limitations caused by tooth loss or reduced dentition can compromise food intake [20]. Oral health directly influences chewing ability. In the present study, the reduction in the number of teeth observed in 78.8% of the participants (≤19 teeth) was associated with a higher chance of frailty in bivariate analysis. However, after adjustment, fair or poor chewing, present in 37% of the older adults evaluated, remained a significant variable. Chewing is a functional activity relevant to both edentulous and dentate individuals, including those who use complete or partial dentures. Perceived chewing ability plays a crucial role in nutrition as it directly influences food selection and nutritional intake. In this same sample, a previous study examined the association between oral health, nutritional status, and frailty [26]; chewing was also associated with frailty, assessed through the FRAIL-BR. Despite the use of two different tools to assess frailty, this result reinforces the essential role of both oral health and nutritional status in the healthy aging process [26]. According to Woo et al. [27], chewing difficulty should be included in primary care screening of geriatric syndromes and chronic diseases. Furthermore, incomplete or non-functional dentition can significantly impair eating, nutritional status, and overall functional health in older adults [27,28]. This scenario can result in malnutrition and excess weight and can increase susceptibility to chronic noncommunicable diseases, impacting longevity and quality of life.

In the present study, both malnutrition and the risk of malnutrition were associated with frailty among older adults. These nutritional conditions lead to a reduction in body fat and muscle mass, predisposing individuals to sarcopenia and triggering the frailty cycle [8,9]. Another aspect of nutritional status that must be considered and has been a matter of concern is obesity. Although BMI was not associated with frailty, obesity is an important factor that, in many cases, particularly among older adults, may indicate sarcopenic obesity. The latter predisposes to frailty and is often underestimated [29]. Identification of these conditions is necessary so that resources can be allocated to appropriate preventive treatment and care.

A possible explanation for these findings may be related to socioeconomic status, which encompasses income and education, as well as the social determinants of health that influence the behavior and environment of older adults unequally [30]. An income of up to one minimum wage can make it difficult to purchase the fresh and varied foods essential for adequate nutrition since they are often more expensive and inaccessible to individuals with limited economic resources [31]. Regarding education, a multicenter study conducted in southern Brazil [32] revealed that individuals with a higher education level tend to access health services more frequently and have a better understanding of nutrition and health.

The association between income and frailty has been widely recognized in the literature [5,33]. Within this context, equity measures aimed at improving access to health services and health promotion are essential to reduce health inequities. The present study provides a unique perspective by assessing the association between the ICVF-20 and health literacy. Health literacy is a modifiable factor that significantly affects people’s quality of life, disease prevention, and promotion of good health [7]. The present study demonstrated an association between low health literacy and frailty. This association can impact the understanding of symptoms, treatment, management of the health condition, maintenance of independence, and the degree of frailty [6,7]. More studies and strategies are needed to improve health literacy, especially among vulnerable groups. Health behaviors and social determinants must be considered for the promotion of good health [6].

Frailty syndrome is a multifactorial disease [4], as demonstrated in the present study, which is associated with socioeconomic factors, clinical factors such as oral health, nutritional status, and health literacy. It is noted that these factors are interconnected but it was not the objective of the present study to analyze how this interaction occurs. However, further studies must address this interaction to understand the mediation effects of each of these factors.

The association between oral health, nutrition, income, health literacy, and social determinants is intrinsic and directly affects frailty syndrome in older adults. Oral and nutritional health are essential factors since missing teeth and oral diseases can compromise chewing ability and, consequently, the intake of adequate nutrients. Tooth loss and oral health outcomes, as well as other health behaviors, are linked to low literacy [6,7,34] which, in turn, is associated with social determinants such as income and education [5,30].

Given the specific care required for older adults and considering that this study was conducted in the PHC setting, the importance of a multidisciplinary approach that includes a nutritionist [35] and dentist [36], among other health professionals, is evident. It is important to note that this study was conducted in a municipality with extensive primary care coverage and a high Human Development Index (HDI) [15]. However, the prevalence of CFV continues to be high. This scenario may be more worrying in other regions of Brazil that lack this infrastructure. The IVCF-20, a screening tool developed and validated in Brasil, proved useful in detecting frailty within PHC and could be applied or adapted to other health systems to support standardized and early identification of vulnerability. A universal approach to early frailty screening may help reduce inequities resulting from socioeconomic disparities, ensuring more equitable and effective care.

These findings have direct implications for clinical practice and public health planning. The identification of modifiable factors—such as poor chewing ability, nutritional status and low health literacy—provides actionable targets for multidisciplinary interventions within Primary Health Care (PHC). Incorporating frailty assessment using the IVCF-20 into routine care can support early detection, individualized follow-up, and timely referral to rehabilitation or social support programs. A multidisciplinary team approach is essential to ensure comprehensive care, expanding the professional perspective beyond disease treatment toward the prevention of frailty. Moreover, developing competencies among health professionals during training to recognize early predictors of frailty can strengthen preventive practices and improve patient outcomes. Implementing health education strategies that promote health, and emphasizing health literacy, oral health, and adequate nutrition may mitigate vulnerability, reduce healthcare costs, and foster health aging. Future studies should evaluate the effectiveness of these interventions in reducing frailty progression and improving quality of life among older people.

One limitation of this study was its observational and cross-sectional design, which did not permit the establishment of causal relationships. In addition, the sample was restricted to users of five BHUs. However, we sought to obtain a diverse profile of these units by including different regions of the municipality. However, the present sample is not representative of the municipality. Since a questionnaire was administered for data collection, the possibility of information bias must be considered. Healthy longevity depends on specific health policies and interventions for prevention and treatment. This study identified associated factors that are crucial for understanding the dynamics of frailty syndrome and the importance of planning specific actions and care to reduce the impact on the public health system.

## 5. Conclusions

This study identified an association between CFV and different determinant factors, including low socioeconomic conditions, compromised nutritional status, poor/fair chewing, and low health literacy. PHC, as the gateway to SUS, plays an essential role in promoting comprehensive care and reducing inequities. Public policies aimed at health promotion and the early identification of the stages of CFV are key to mitigating risks, preventing negative outcomes, and ensuring a more effective patient-centered approach. The present findings highlight the multifactorial aspect of CFV in older adults, contributing significantly to the development of strategies that favor healthy and sustainable aging.

## Figures and Tables

**Table 1:** Sociodemographic characterization of the sample studied (n = 211), Jundiaí, SP, Brazil, 2023.

Variables		n (%)	95%CI
Sex			
	Female	152 (72)	64.6–78.3
	Male	59 (28)	21.7–35.4
Age			
	≤69 years	111 (52.6)	46.4–60.2
	≥70 years	100 (47.4)	39.8–53.6
Skin color			
	White	159 (75.4)	67.2–79.7
	Black/brown	52 (24.6)	20.3–32.8
Marital status			
	Single	103 (48.8)	41.3–56.3
	Married	108 (51.2)	43.8–58.7
Education			
	Incomplete elementary school	123 (58.3)	53.1–65.6
	Complete elementary school	40 (19)	14.1–24.3
	High school or higher	48 (22.7)	16.3–28.8
Income			
	Up to 1 minimum wage	83 (39.3)	33.5–46.7
	2 or more minimum wages	128 (60.7)	53.3–66.5
Current smoker			
	Yes	18 (8.5)	5.2–12.2
	No	193 (91.5)	87.8–94.8
Former smoker			
	Yes	96 (45.5)	39.8–51.5
	No	115 (54.5)	48.5–60.2
Current daily alcohol consumption			
	Yes	6 (2.8)	0.5–5.2
	No	205 (97.2)	94.8–99.5
Past daily alcohol consumption			
	Yes	36 (17.1)	10.9–21.6
	No	175 (82.9)	78.4–89.1
Medication use			
	No medication	23 (10.9)	5.4–14.4
	1 to 3 medications	51 (24.2)	15.6–29.0
	4 or more medications	137 (64.9)	59.9–74.5
Health problems			
	Yes	189 (89.6)	85.0–93.2
	No	22 (10.4)	6.8–15.0
Type of health problem			
	Diabetes	12 (5.7)	2.8–8.5
	Hypertension	72 (34.1)	28.9–41.4
	Both	69 (32.7)	26.1–38.4
	Others	58 (27.5)	20.9–34.6
History of COVID-19			
	Yes	77 (36.5)	29.4–43.1
	No	134 (63.5)	56.9–70.6
Hospitalization in the last year			
	Yes	39 (18.5)	14.2–24.0
	No	172 (81.5)	76.0–85.8
Frequency of use of Basic Health Unit services			
	1x per year	74 (35.1)	86.8–95.3
	2x or more per year	137 (64.9)	4.7–13.2
Number of teeth			
	≤19	166 (78.7)	71.7–83.9
	≥20	45 (21.3)	16.1–28.3
Chewing			
	Good	133 (63)	57.3–69.5
	Fair	43 (20.4)	13.9–25.6
	Poor	35 (16.6)	11.8–22.3
Body mass index (BMI)			
	Low weight	29 (13.7)	9.0–18.2
	Eutrophy	80 (37.9)	28.6–42.2
	Pre-obesity	31 (14.7)	9.4–20.3
	Obesity	71 (33.6)	28.6–41.7
Participation in physical activities offered			
	Yes	78 (37)	31.3–43.6
	No	133 (63)	56.4–68.7
10-Point Cognitive Screener (10-CS)			
	Normal	135 (64)	56.4–72.0
	Possible impairment	57 (27)	21.4–34.2
	Probable impairment	19 (9)	4.7–12.5
Mini Nutritional Assessment (MAN)			
	Normal nutritional status	136 (64.5)	55.9–69.6
	Nutritional risk or malnourished	75 (35.5)	30.4–44.1
Health literacy (HLS-14)			
	High literacy	108 (51.2)	43.3–57.6
	Low literacy	103 (48.8)	42.4–56.6
SARC-F+CC			
	No sarcopenia risk	174 (82.5)	75.2–87.5
	Sarcopenia risk	37 (17.5)	12.5–24.8
Clinical-Functional Vulnerability Index (IVCF-20)			
	Low clinical-functional vulnerability	118 (55.9)	45.0-59.9
	Medium clinical-functional vulnerability	73 (34.6)	30.7–43.6
	High clinical-functional vulnerability	20 (9.5)	6.3–15.1

Descriptive analysis: absolute frequency and percentage.

**Table 2:** Distribution of items of the IVCF-20 administered to community-dwelling older adults in primary care (n = 211). Jundiaí, SP, Brazil, 2023.

Components of the Clinical-Functional Vulnerability Index-20				n	%
Age		60 to 74 years		154	73.0
		75 to 84 years		48	22.7
		≥85 years		9	4.3
Self-perception of health	Health compared to other people of your age	Excellent/very good/good		171	81.0
		Fair or poor		40	19.0
Activities of daily living (ADL)	ADL (instrumental)	Does no longer go shopping	Yes	16	7.6
			No	195	92.4
		Does no longer control finances	Yes	16	7.6
			No	195	92.4
		Does no longer do small household chores	Yes	13	6.2
			No	198	93.8
	ADL (basic)	Does no longer take a bath alone	Yes	2	0.9
			No	209	99.1
Cognition	Becoming forgetful		Yes	67	31.8
			No	144	68.2
	Forgetfulness worsened in the last month		Yes	45	21.3
			No	166	78.7
	Forgetfulness prevents the execution of ADL		Yes	23	10.9
			No	188	89.1
Mood	Discouragement, sadness or hopelessness in the last month		Yes	103	48.8
			No	108	51.2
	Loss of interest or pleasure in previously pleasurable activities in the last month		Yes	70	33.2
			No	141	66.8
Mobility	Reach, grip and pinch	Unable to raise arms above shoulder	Yes	10	4.7
			No	201	95.3
		Unable to raise arms above shoulder	Yes	11	5.2
			No	200	94.8
	Aerobic and/or muscle capacity	Unintentional weight loss. BMI <22 kg/m^2^, calf circumference <31 cm, or gait speed (4 m) >5 s	Yes	47	22.3
			No	164	77.7
	Gait	Difficulty walking that can impede ADL	Yes	33	15.6
			No	178	84.4
		Two or more falls in the last year	Yes	23	10.9
			No	188	89.1
	Urinary/bowel continence	Unintentional urine or feces loss	Yes	63	29.9
			No	148	70.1
Communication	Vision	Visual impairment that prevents the execution of activities	Yes	15	7.1
			No	196	92.9
	Hearing	Hearing impairment that prevents the execution of activities	Yes	7	3.3
			No	204	96.7
Multiple comorbidities	Polypathology Polypharmacy	≥5 chronic diseases ≥5 daily medications or hospitalization in the last 6 months	Yes	85	40.3
			No	126	59.7

**Table 3:** Bivariate analysis between independent variables and clinical-functional vulnerability of older adults in primary care (n = 211). Jundiaí, SP, Brazil, 2023.

Variables		Low CFV		Medium or high CFV		*p*
		n	%	n	%	
Sex	Female	81	53.3	71	46.7	0.216
	Male	37	62.7	22	37.3	
Age	≤69 years	74	66.7	37	33.3	0.001
	≥70 years	44	44	56	56	
Skin color	White	97	61	62	39	0.009
	Black/brown	21	40.4	31	59.6	
Marital status	Single	50	48.5	53	51.5	0.035
	Married	68	63	40	37	
Education	Incomplete elementary school	55	44.7	68	55.3	0.001
	Complete elementary school	28	70	12	30	
	High school or higher	35	72.9	13	27.1	
Income	Up to 1 minimum wage	36	43.4	47	56.6	0.003
	2 or more minimum wages	82	64.1	46	35.9	
Current smoker	Yes	12	66.7	6	33.3	0.337
	No	106	54.9	87	45.1	
Former smoker	Yes	55	57.3	41	42.7	0.715
	No	63	54.8	52	45.2	
Current daily alcohol consumption	Yes	4	66.7	2	33.3	0.591
	No	114	55.6	91	44.4	
Past daily alcohol consumption	Yes	17	47.2	19	52.8	0.248
	No	101	57.7	74	42.3	
Medication use	Up to 3 medications	59	79.7	15	20.3	<0.001
	4 or more medications	59	43.1	78	56.9	
Health problems	Yes	98	51.9	91	48.1	<0.001
	No	20	90.9	2	9.1	
History of COVID-19	Yes	48	62.3	29	37.7	0.155
	No	70	52.2	64	47.8	
Frequency of use of Basic Health Units	1x per year	102	53.4	89	46.6	0.023
	2x or more per year	16	80.0	4	20.0	
Hospitalization in the last year	Yes	16	41.0	23	59.0	0.380
	No	102	59.3	70	40.7	
Physical activity	Yes	48	61.5	30	38.5	0.208
	No	70	52.6	63	47.4	
Number of teeth	≤19	84	50.6	82	49.4	0.003
	≥20	34	75.6	11	24.4	
Chewing	Good	92	69.2	41	30.8	<0.001
	Fair	17	39.5	26	60.5	
	Poor	9	25.7	26	74.3	
Body mass index	Low weight	15	51.7	14	48.3	0.536
	Eutrophy	47	58.8	33	41.2	
	Pre-obesity	20	64.5	11	35.5	
	Obesity	36	50.7	35	49.3	
SARC-F+CC	No sarcopenia risk	105	60.3	69	39.7	0.005
	Sarcopenia risk	13	35.1	24	64.9	
10–Point Cognitive Screener (10-CS)	Normal	89	65.9	46	34.1	<0.001
	Possible impairment	24	42.1	33	57.9	
	Probable impairment	5	26.3	14	73.7	
Mini Nutritional assessment (MNA)	Normal nutritional status	91	66.9	45	33.1	<0.001
	Risk of malnutrition or malnourished	27	36	48	64	
Health literacy (HLS-14)	High literacy	70	64.8	38	35.2	0.008
	Low literacy	48	46.6	55	53.4	

Chi-squared test, *p* < 0.05.

**Table 4:** Factors associated with clinical-functional vulnerability after multiple binary logistic regression in community-dwelling older adults treated in primary care (n = 211). Jundiaí, SP, Brazil, 2023.

Variables		Unadjusted			Adjusted		
		OR	95%CI	*p*	OR	95%CI	*p*
Age	≥70 years	2.55	1.45–4.44	0.001			
	≤69 years	1					
Skin color	Black/brown	2.31	1.21–4.37	0.010			
	White	1					
Marital status	Single	1.80	1.04–3.12	0.036			
	Married	1					
Education	Incomplete elementary school	3.33	1.60–6.90	0.001			
	Complete elementary school	1.15	0.45–2.92	0.763			
	Complete high school	1					
Income	Up to 1 minimum wage	2.33	1.32–4.09	0.003	1.90	1.02–3.55	0.043
	2 or more minimum wages	1			1		
Medication use	4 or more medications	5.20	2.68–10.06	<0.001			
	Up to 3 medications	1					
Health problems	Yes	9.28	2.11–40.84	0.003			
	No	1					
History of COVID-19	Yes	0.66	0.37–1.17	0.156			
	No	1					
Frequency of use of Basic Health Units	1x per year	3.49	1.12–10.82	0.030			
	2x or more per year	1					
Hospitalization in the last year	Yes	2.09	1.03–4.24	0.040			
	No	1					
Number of teeth	≤19	3.01	1.43–6.35	0.004			
	≥20	1					
Chewing	Poor	6.48	2.79–15.05	<0.001	5.18	2.13–12.63	<0.001
	Fair	3.43	1.68–7.00	0.001	2.36	1.10–5.05	0.027
	Good	1			1		
SARC-F+CC	No sarcopenia risk	2.80	1.34–5.88	0.006			
	Sarcopenia risk	1					
10-Point Cognitive Screener (10-CS)	Probable impairment	5.41	1.83–15.97	0.002			
	Possible impairment	2.66	1.41–5.02	0.003			
	Normal	1					
Mini Nutritional Assessment (MNA)	Risk of malnutrition or malnourished	3.59	1.99–6.49	<0.001	2.36	1.23–5.52	0.009
	Normal nutritional status	1			1		
Health literacy (HLS-14)	Low literacy	2.11	1.21–3.67	0.008	1.86	1.09–3.45	0.047
	High literacy	1			1		

Unadjusted and adjusted odds ratio (OR), 95% confidence interval (95%CI) and *p*-value (*p* < 0.05).

## Data Availability

All anonymized data supporting the findings of this study are available from the Open Science Framework (OSF) at https://osf.io/rg75y/?view_only=3f6ea18761ff4e8ebc309568c3334e23 (accessed on 13 October 2025).
